# Gastrointestinal Symptoms and Irritable Bowel Syndrome Are Associated With Female Sex and Smoking in the General Population and With Unemployment in Men

**DOI:** 10.3389/fmed.2021.646658

**Published:** 2021-09-01

**Authors:** Daniel Nilsson, Bodil Ohlsson

**Affiliations:** Department of Internal Medicine, Skåne University Hospital, Lund University, Malmö, Sweden

**Keywords:** irritable bowel syndrome, gastrointestinal symptoms, lifestyle habits, population-based, sociodemography, smoking, snuff, unemployment

## Abstract

**Background:** The influence of daily life exposure on the gastrointestinal tract is not fully understood. This study aimed to examine associations between functional gastrointestinal symptoms and sociodemographic status and lifestyle habits in the general population.

**Methods:** The Malmö Offspring Study (MOS) included 2,648 participants from the general population who had answered a questionnaire about sociodemographic status, lifestyle habits, medical health, and self-reported irritable bowel syndrome (IBS). The visual analog scale for IBS (VAS-IBS) was completed to assess gastrointestinal symptoms the past 2 weeks. Subjects with organic gastrointestinal diseases were excluded. Presence of self-reported IBS and gastrointestinal symptoms the past 2 weeks were used as dependent variables to study the associations with age, sex, body mass index, education, occupation, marital status, smoking, snuff using, alcohol drinking frequency, alcohol amount per drinking occasion, physical activity at work, and physical activity during leisure time, using logistic regression and generalized linear model.

**Results:** Self-reported IBS was associated with gastrointestinal symptoms the past 2 weeks (*p* < 0.001). There was an association between IBS and female sex (*p* < 0.001), former smoking (*p* < 0.001), present smoking (*p* < 0.001), and an inverse association with drinking 3–4 standard glasses per occasion (*p* = 0.038). Gastrointestinal symptoms were associated with age 50–59 years (*p* = 0.009), ≥60 years (*p* = 0.004), female sex (*p* < 0.001), studying (*p* = 0.036), unemployment (*p* = 0.009), former smoking (*p* = 0.001), and present smoking (*p* = 0.012). In men, IBS was associated with middle-age and both IBS and gastrointestinal symptoms were associated with unemployment (*p* < 0.001 and *p* = 0.001, respectively). In women, IBS was associated with present smoking (*p* = 0.022), and gastrointestinal symptoms were associated with former smoking and inversely associated with higher age (*p* = 0.006) and intermediate physical activity at work (*p* = 0.008). No associations were found with BMI, education, marital status, or snuff using.

**Conclusion:** Self-reported IBS in the general population shows strongest association with female sex and smoking, whereas gastrointestinal symptoms also are associated with unemployment and inversely associated with higher age. In men, both IBS and gastrointestinal symptoms are associated with unemployment. In women, both IBS and gastrointestinal symptoms are associated with smoking, whereas symptoms are inversely associated with higher age and intermediate physical activity.

## Introduction

The mechanism behind the functional gastrointestinal disorders (FGIDs) is unclear, and many factors such as low-grade inflammation, dysbiosis of the gut microbiota, and psychological factors are discussed as etiological factors (1, 2). Irritable bowel syndrome (IBS) is the most common of the FGIDs (2). The prevalence of IBS varies between different versions of the Rome criteria but may occur in around 5–11% of the population (3, 4). Besides complaining of the symptoms, the disease is also a common reason for staying at home from work or school (5, 6). The symptoms are treated with dietary advice, psychological treatment, and drugs to relief symptoms (2). Although IBS is the most common disease at a Department of Gastroenterology, most of the patients are handled at primary healthcare centers or do not visit any physician at all (1). Still, most of the research in this entity is performed on patients from tertiary care centers.

Regarding the associations between IBS and sociodemographic factors and lifestyle habits, the results are contradictory depending on country; sample composition and sample size; and Rome criteria used. It is well-described that IBS is associated with female sex (2, 7), whereas there has not been found any significant difference in IBS prevalence regarding age and socioeconomic factors in a large meta-analysis (7). Female patients with IBS in Iran were more often single and being unemployed than patients without IBS (8), whereas married or single subjects in a Bulgarian population-based study had lower IBS prevalence than those only living in a relationship (9). An inverse association between IBS and body mass index (BMI) was observed in men in one large French study (10), with no association between IBS and BMI when calculations were performed with both sexes together (9–11).

In a systematic review, a weak association between smoking and FGIDs were observed; the association was varying dependent on different criteria for FGID (12). In contrast, we have previously shown in a population-based study of middle-age and elder subjects, that smoking was associated with several functional gastrointestinal (GI) symptoms (13). The role of alcohol has been even more difficult to determine, showing no association (14, 15), a tendency toward less symptoms with a moderate alcohol intake (13), or aggravated symptoms after high intake of alcohol (12, 15). On the other hand, a higher prevalence of IBS was found in non-users of alcohol in a population-based Bulgarian study (9). The role of snuff, a Swedish variant of tobacco, has not been examined regarding its association with FGIDs. Higher physical activity was associated with less GI symptoms in middle-age and elder Swedish subjects from the general population (11).

Our hypothesis was that smoking and snuff using have similar adverse impact on GI symptoms and that higher alcohol consumption, living alone, and lower BMI, education, and physical activity are all associated with more GI symptoms. The aim in this population-based cohort was to examine association of self-reported IBS and GI symptoms the past 2 weeks with sociodemographic factors and lifestyle habits.

## Materials and Methods

The study was conducted according to the Declaration of Helsinki and approved by the Regional Ethics Review Board in Lund (2012/594). Written, informed consent was given by all participants prior to study start.

### Study Participants

**Figure 1:**
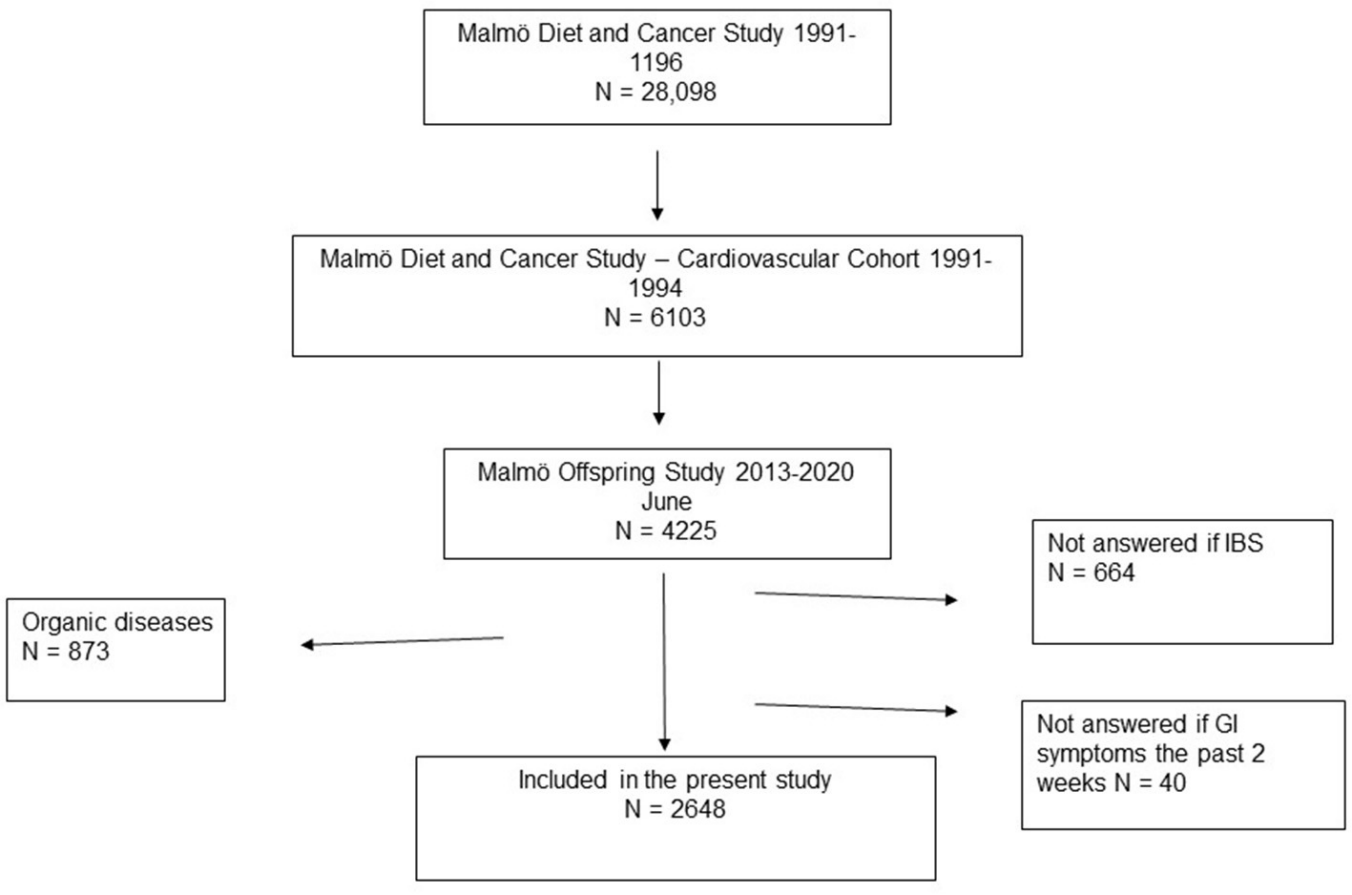
The diagram shows the origin of the participants. Malmö Diet and Cancer Study invited everyone living in Malmö between 1991 and 1996 and were born between 1923 and 1950. A total of 28,098 patients completed all tests. A subcategory, the cardiovascular cohort, was extracted from those participants, containing a total of 6,103 randomly selected patients who participated between the years 1991 and 1994. Malmö Offspring Study (MOS) was a population-based cohort consisting of children and grandchildren to someone who was included in the MDC-CC. This study cohort included those from MOS who had answered the following two questions: “Do you several times a month suffer from abdominal pain and irregular bowel habits known as IBS?” and “Have you experienced bowel symptoms during the past 2 weeks?” Participants with any organic gastrointestinal diseases (celiac disease, Crohn's disease, ulcerative colitis, lactose intolerance, reflux, and ulcer) were excluded.

The Malmö Diet and Cancer Study (MDCS) invited all subjects born between 1923 and 1950, and living in Malmö between 1991 and 1996, to take part in the study. A total of 28,098 patients completed all tests (16). Later, 6,103 individuals were re-examined for assessing cardiovascular risk factors and constitutes the Malmö Diet and Cancer Cardiovascular Cohort (MDC-CC) (17). The Malmö Offspring Study (MOS) is a population-based cohort consisting of children and grandchildren to participants in the MDC-CC (18). Subjects were invited to an anthropometric and clinical examination including a measurement of weight and height. From the total of 4225 participants included in June 2020 (inclusion rate 47%), 1,577 were excluded due to organic bowel diseases such as celiac disease, Crohn's disease, ulcerative colitis, lactose intolerance, reflux, and/or ulcer, or due to that they had not answered the questions regarding GI symptoms. Thus, 2,648 individuals were finally included in the present study (Figure 1).

### Study Questionnaire

The MOS included a web-based survey with questions regarding living conditions, education, occupation, tobacco, alcohol, physical activity, medical health, heredity, and medication. The participants were interpreted as having self-reported IBS according to Rome III criteria (19) if they answered “yes” to the following question: “Have you several times during a month suffered from abdominal pain related to irregular bowel habits which is called IBS?”

### Visual Analog Scale for Irritable Bowel Syndrome

The participants were asked: “Have you experienced GI symptoms the past 2 weeks?” If they answered “yes” to this question, they were encouraged to complete the validated visual analog scale for irritable bowel syndrome (VAS-IBS) regarding GI symptoms the last 2 weeks (20). The VAS-IBS assesses the degree of abdominal pain, diarrhea, constipation, bloating and flatulence, vomiting and nausea, intestinal symptom's influence on daily life, and psychological well-being on scales ranging from 0 to 100 mm, where a higher value means more symptoms. The scales were inverted from the original version (20).

### Data Categorization and Modifications

Age was divided into <30, 30–39, 40–49, 50–59, or ≥ 60 years. Sex was divided into men and women. BMI was grouped according to the World Health Organization (WHO) standard into normal or underweight (<25 kg/m^2^), overweight (25.0–29.9 kg/m^2^), and obesity (≥ 30 kg/m^2^) (21). Education was divided into primary school or less, secondary school, and higher education. Marital status was divided into those who lived alone, those who lived together with someone, and a separate group for others. Occupation was divided into working, studying, sick leave, unemployment, retirement, or other, where the category other was not further defined. Participants who had chosen several alternatives were classified as missing values. Smoking and snuff using were grouped into never, former, or present users. Drinking frequency last year was separated into ≤ 1 time/month, 2–4 times per month, 2–3 times/week, ≥ 4 times/week. Drinking amount per occasion was defined with standard glasses (12 grams alcohol) and divided into 1–2, 3–4, 5–6, 7–9, or ≥ 10 glasses. Physical activity at work last year was separated into light (mostly stationary to standing with light muscle activity, e.g., eating and washing-up), intermediate (muscle activity with intermediate intensity, e.g., walking and cleaning), and hard (heavy muscle activity, e.g., gardening). Physical activity during leisure time last year was grouped into sedentary to moderate (reading, watching tv, biking, and moving without sweating), or training regularly (exercise > 30 min twice a week or participating in sports).

### Statistical Analysis

IBM SPSS (Statistical Product and Service Solutions) version 26 for Windows was used to perform the statistical calculations. Data were analyzed with KS-test (Kolmogorov-Smirnov) and histograms to test for normal distribution for the scaled variables. None of the parameters followed a normal distribution and data is therefore presented as median and interquartiles or number and percentages. Pearson Chi-square test was used for calculations on dichotomous variables to determine differences between sexes and between the study groups IBS/no IBS and GI symptoms the past 2 weeks/no GI symptoms.

Logistic regression was used to examine the associations between self-reported IBS and GI symptoms with sociodemographic status and lifestyle factors, with IBS or GI symptoms the past 2 weeks as dependent variables and age, sex, BMI, education, occupation, marital status, smoking, snuff using, drinking frequency past year, drinking amount per occasion, physical activity at work last year, and physical activity during leisure time last year as independent variables. Crude Odds Ratio (OR) and 95% confidence interval (CI) were calculated for each variable. Adjusted OR were then calculated, adjusted for the significant parameters in the crude calculations, i.e., age, sex, smoking, drinking frequency past year and drinking amount per occasion for IBS and age, sex, BMI, education, occupation, smoking, and drinking frequency for GI symptoms. The lowest group, men (sex), or those who never used substances (tobacco or snuff), were used as reference values. To test for sex interaction (i), a multiplicative variable (e.g., sex ^x^ age groups) was added in the adjusted models with all variables. Separate logistic regressions based on sex were conducted, first to calculate crude OR and then adjusted for age and occupation in men and smoking and snuff for women concerning the presence of IBS, and adjusted for age, BMI, and occupation in men and age, occupation, smoking, and physical activity at work in women concerning the presence of GI symptoms.

Generalized linear model was used to examine the association between self-reported IBS and GI symptoms according to the VAS-IBS, with previously found significant parameters as predictors (sex, smoking, drinking frequency, and drinking amount per occasion), and between specific GI symptoms according to the VAS-IBS and age, sex, occupation and smoking, which were significantly associated with GI symptoms in the logistic regression. Values are presented as β (beta-value) and 95% CI. *P* < 0.05 considered as statistically significant.

## Results

### Population Characteristics

Of the 2,648 participants included (1,391 women [52.5%]), 316 participants (11.9%) had self-reported IBS, and 459 participants (17.3%) had experienced GI symptoms in the past 2 weeks. Those with IBS had more symptoms than those without IBS (Table 1), and there was a strong association between all individual symptoms and self-reported IBS (Table 2). The participants with IBS were 43.38 (29.34–52.52) years old and had a BMI of 24.54 (22.27–27.63) kg/m^2^, and those without IBS were 45.59 (29.28–54.86) years old and had a BMI of 25.22 (22.76–28.38) kg/m^2^ (*p* = 0.128 and *p* = 0.011, respectively). The age in those who had GI symptoms the past 2 weeks was 40.50 (27.58–51.22) years and the BMI was 24.30 (22.13–27.82) kg/m^2^, in comparison to 46.18 (29.75–55.15) years and 25.25 (22.84–28.39) kg/m^2^ in those without symptoms (*p* < 0.001 and *p* = 0.001, respectively). Subjects with self-reported IBS were more prevalent in lower age groups (*p* = 0.005), were more often women (*p* < 0.001) and smokers (*p* = 0.001) and drank alcohol less often (*p* = 0.014) than those without IBS (Table 3). Subjects with GI symptoms the past 2 weeks were more prevalent in lower age groups (*p* < 0.001), were more often women (*p* < 0.001), unemployed or sick (*p* = 0.003), and smokers (*p* = 0.008), and had lower BMI (*p* = 0.015) and education degree (*p* = 0.05), than those without GI symptoms (Table 4). The 297 present snuff users were most often found in the group of former smokers (*n* = 124) and least often in the group of present smokers (*n* = 72) (*p* < 0.001).

**Table 1:** Distribution of gastrointestinal symptoms.

**Symptoms**	**IBS (***n*** = 2,648)**			**GI symptoms past 2 weeks (***n*** = 2,648)**	
	**No (***n*** = 2,332)**	**Yes (***n*** = 316)**	***P*** **-value**	**No (***n*** = 2,189)**	**Yes (***n*** = 459)**
Abdominal pain	10 (1–27)	40 (14–60)	0.029	–	21 (5–50)
Missing	2,093	113			42
Diarrhea	10 (1–39)	31 (8–62)	0.019	–	21 (4–56)
Missing	2,091	118			45
Constipation	4 (0–23)	32 (4–60)	0.058	–	10 (1–50)
Missing	2,093	128			57
Bloating and flatulence	20 (4–50)	60 (37–74)	<0.001	–	45 (18–68)
Missing	2,088	108			32
Vomiting and nausea	3 (0–13)	5 (0–38)	0.301	–	4 (0–26)
Missing	2,096	131			63
Influence on daily life	15 (2–49)	50 (19–68)	0.009	–	30 (10–60)
Missing	2,080	105			21
Psychological well-being	15 (5–30)	25 (10–56)	<0.001	15 (5–29)	22 (7–50)
Missing	152	16		149	19

*GI, gastrointestinal; IBS, self-reported irritable bowel syndrome. Symptoms the past 2 weeks were assessed by the visual analog scale for irritable bowel syndrome (VAS-IBS) where 0 mm means no symptoms and 100 mm means maximal symptoms (22). Values are presented as median and 25th−75th percentile. Participants who answered “No” to question about having any GI symptoms the past 2 weeks were only supposed to answer the last question (psychological well-being). Mann-Whitney U-test. P < 0.05 was considered statistically significant*.

**Table 2:** Associations between gastrointestinal symptoms the past 2 weeks and having IBS.

**Symptoms**	**β**	**95% CI**	***P*** **-value**
Abdominal pain	22.332	15.017–29.647	<0.001
Diarrhea	29.016	21.276–36.757	<0.001
Constipation	10.623	2.767–18.479	0.008
Bloating and flatulence	22.721	14.820–30.622	<0.001
Vomiting and nausea	14.918	7.767–22.069	<0.001
Influence on daily life	28.531	20.541–36.521	<0.001
Psychological well-being	20.579	18.200–22.957	<0.001

*β, beta-value; CI, confidence interval. Symptoms the past 2 weeks were assessed by the visual analog scale for irritable bowel syndrome (VAS-IBS) where 0 mm means no symptoms and 100 mm means maximal symptoms (20)*.

*Generalized linear model, adjusted for sex, smoking, drinking frequency, and drinking amount per occasion. P < 0.05 was considered as statistically significant*.

**Table 3:** Association between sociodemographic factors, lifestyle habits, and IBS.

**Variables**	**No IBS 2332 (88.1)**	**IBS 316 (11.9)**	**Crude OR and 95% CI**	***P*** **-value**	**OR and 95% CI**	***P*** **-value**
**Age (years)**						
<30 (reference)	614 (26.3)	83 (26.3)	1.000		1.000	
30–39	354 (15.2)	60 (19.0)	1.254 (0.877–1.792)	0.214	1.343 (0.901–1.999)	0.147
40–49	433 (18.6)	75 (23.7)	1.281 (0.916–1.792)	0.148	1.365 (0.925–2.013)	0.117
50–59	677 (29.0)	63 (19.9)	0.688 (0.487–0.972)	0.034	0.730 (0.489–1.090)	0.124
≥60	254 (10.9)	35 (11.1)	1.019 (0.669–1.553)	0.929	0.920 (0.556–1.523)	0.746
**Sex**						
Men (reference)	1,163 (49.9)	94 (29.7)	1.000		1.000	
Women	1,169 (50.1)	222 (70.3)	2.350 (1.822–3.030)	<0.001	2.156 (1.620–2.868)	<0.001
**BMI (kg/m^2^)**						
<25 (reference)	1,120 (48.0)	173 (54.7)	1.000			
25.0–29.9	807 (34.6)	98 (31.0)	0.786 (0.604–1.024)	0.074		
≥ 30	405 (17.4)	45 (14.2)	0.719 (0.508–1.018)	0.063		
**Education**						
Primary school or less (reference)	141 (6.0)	20 (6.3)	1.00			
Secondary school	1,267 (54.3)	164 (51.9)	0.913 (0.556–1.498)	0.718		
Higher education	916 (39.3)	128 (40.5)	0.985 (0.595–1.630)	0.954		
*Missing*	8 (0.3)	4 (1.3)				
**Occupation**						
Working (reference)	1,627 (69.8)	204 (64.6)	1.000			
Studying	153 (6.6)	21 (6.6)	1.095 (0.678–1.767)	0.711		
Sick leave	20 (0.9)	6 (1.9)	2.393 (0.950–6.027)	0.064		
Unemployment	56 (2.4)	10 (3.2)	1.424 (0.715–2.835)	0.314		
Retirement	53 (2.3)	5 (1.6)	0.752 (0.297–1.904)	0.548		
Other	46 (2.0)	8 (2.5)	1.387 (0.646–2.980)	0.402		
*Missing*	377 (16.2)	62 (19.6)				
**Marital status**						
Living alone (reference)	583 (25.0)	86 (27.2)	1.000			
Living together	1,554 (66.6)	200 (63.3)	0.872 (0.666–1.143)	0.322		
Other	190 (8.1)	30 (9.5)	1.070 (0.685–1.673)	0.765		
*Missing*	5 (0.2)	0 (0.0)				
**Smoking**						
Never (reference)	1,450 (62.2)	163 (51.6)	1.000		1.000	
Former	559 (24.0)	92 (29.1)	1.464 (1.114–1.925)	0.006	1.744 (1.285–2.367)	<0.001
Present	319 (13.7)	61 (19.3)	1.701 (1.237–2.338)	0.001	1.925 (1.352–2.742)	<0.001
*Missing*	4 (0.2)	0 (0.0)				
**Snuff using**						
Never (reference)	1,848 (79.2)	250 (79.1)	1.000			
Former	194 (8.3)	29 (9.2)	1.105 (0.732–1.668)	0.635		
Present	264 (11.3)	33 (10.4)	0.924 (0.629–1.358)	0.688		
*Missing*	26 (1.1)	4 (1.3)				
**Drinking frequency past year**						
Never (reference)	139 (6.0)	28 (8.9)	1.000		1.000	
≤1 time/month	474 (20.3)	71 (22.5)	0.744 (0.462–1.197)	0.223	1.059 (0.772–1.452)	0.722
2–4 times/month	974 (41.8)	140 (44.3)	0.714 (0.458–1.111)	0.135	0.841 (0.581–1.218)	0.359
2–3 times/week	647 (27.7)	72 (22.8)	0.552 (0.344–0.887)	0.014	0.352 (0.123–1.006)	0.051
≥ 4 times/week	90 (3.9)	4 (1.3)	0.221 (0.075–0.650)	0.006	–	
*Missing*	8 (0.3)	1 (0.3)				
**Drinking amount/occasion (glasses)**						
1–2 (reference)	1,189 (51.0)	175 (55.4)	1.000		1.000	
3–4	659 (28.3)	67 (21.2)	0.691 (0.513–0.930)	0.015	0.716 (0.522–0.982)	0.038
5–6	221 (9.5)	31 (9.8)	0.953 (0.634–1.433)	0.817	1.036 (0.659–1.628)	0.878
7–9	90 (3.9)	10 (3.2)	0.755 (0.385–1.479)	0.412	0.929 (0.448–1.927)	0.843
≥ 10	23 (1.0)	4 (1.3)	1.182 (0.404–3.457	0.761	1.661 (0.535–5.162)	0.380
*Missing*	150 (6.4)	29 (9.2)				
**Physical activity at work**						
Light (reference)	1,349 (57.8)	178 (56.3)	1.000			
Intermediate	581 (24.9)	77 (24.4)	1.004 (0.756–1.335)	0.976		
Hard	323 (13.9)	42 (13.3)	0.985 (0.689–1.409)	0.936		
*Missing*	79 (3.4)	19 (6.0)				
**Physical activity during leisure time**						
Sedentary to moderate (reference)	373 (16.0)	60 (19.0)	1.000			
Training regularly	876 (37.6)	121 (38.3)	0.859 (0.616–1.197)	0.369		
*Missing*	1,083 (46.4)	135 (42.7)				

*IBS, irritable bowel syndrome; BMI, body mass index; OR, odds ratio; CI, confidence interval. Physical activity at work was divided into light (mostly sitting), intermediate (walking, doing laundry), and hard (gardening, heavy lifting). Physical activity during leisure time was divided into sedentary to moderate (mostly sitting, moving without sweating) and training regularly (> 30 min twice a week). Prevalence of variables was given as number and percentage. Logistic regression adjusted for statistically significant variables found in crude OR (age, sex, smoking, drinking frequency past year, and drinking amount per occasion). P < 0.05 was considered statistically significant*.

**Table 4:** Association between sociodemographic status, lifestyle habits, and GI symptoms past 2 weeks.

**Variables**	**No Symptoms 2189 (82.7)**	**Symptoms 459 (17.3)**	**Crude OR and 95% CI**	***P*** **-value**	**OR and 95% CI**	***P*** **-value**
**Age (years)**						
<30 (reference)	555 (25.4)	142 (30.9)	1.00		1.000	
30–39	330 (15.1)	84 (18.3)	0.995 (0.735–1.346)	0.973	1.105 (0.757–1.612)	0.605
40–49	403 (18.4)	105 (22.9)	1.018 (0.767–1.351)	0.900	1.099 (0.756–1.596)	0.622
50–59	643 (29.4)	97 (21.1)	0.590 (0.445–0.782)	<0.001	0.603 (0.412–0.883)	0.009
≥ 60	258 (11.8)	31 (6.8)	0.470 (0.310–0.712)	<0.001	0.384(0.202–0.733)	0.004
**Sex**						
Men (reference)	1,099 (50.2)	158 (34.4)	1.00		1.000	
Women	1,090 (49.8)	301 (65.6)	1.921 (1.557–2.370)	<0.001	1.770 (1.387–2.259)	<0.001
**BMI (kg/m^2^)**						
<25 (reference)	1,043 (47.6)	250 (54.5)	1.00		1.000	
25.0–29.9	773 (35.3)	132 (28.8)	0.712 (0.566–0.897)	0.004	0.899 (0.688–1.176)	0.437
≥ 30	373 (17.0)	77 (16.8)	0.861 (0.650–1.141)	0.298	1.025 (0.726–1.446)	0.899
**Education**						
Primary school (reference)	141 (6.4)	20 (4.4)	1.00		1.000	
Secondary school	1,196 (54.6)	235 (51.2)	1.385 (0.850–2.258)	0.191	1.483 (0.799–2.750)	0.211
Higher education	844 (38.6)	200 (43.6)	1.671 (1.020–2.735)	0.041	1.774 (0.942–3.340)	0.076
*Missing*	8 (0.4)	4 (0.9)				
**Occupation**						
Working (reference)	1,536 (70.2)	295 (64.3)	1.00		1.000	
Studying	132 (6.0)	42 (9.2)	1.657 (1.146–2.396)	0.007	1.597 (1.031–2.473)	0.036
Sick leave	20 (0.9)	6 (1.3)	1.562 (0.622–3.923)	0.342	1.473 (0.565–3.839)	0.428
Unemployment	47 (2.1)	19 (4.1)	2.105 (1.218–3.638)	0.008	2.143 (1.207–3.804)	0.009
Retirement	54 (2.5)	4 (0.9)	0.386 (0.139–1.073)	0.068	0.901 (0.281–2.892)	0.861
Other	45 (2.1)	9 (2.0)	1.041 (0.504–2.153)	0.913	0.960 (0.457–2.020)	0.915
*Missing*	355 (16.2)	84 (18.3)				
**Marital status**						
Living alone (reference)	541 (24.7)	128 (27.9)	1.00			
Living together	1,471 (67.2)	283 (61.7)	0.813 (0.646–1.024)	0.079		
Other	173 (7.9)	47 (10.2)	1.148 (0.789–1.672)	0.471		
*Missing*	4 (0.2)	1 (0.2)				
**Smoking**						
Never (reference)	1,361 (62.2)	252 (54.9)	1.00		1.000	
Former	526 (24.0)	125 (27.2)	1.283 (1.013–1.626)	0.039	1.518 (1.148–2.007)	0.003
Present	298 (13.6)	82 (17.9)	1.486 (1.124–1.964)	0.005	1.479 (1.055–2.072)	0.023
*Missing*	4 (0.2)	0 (0.0)				
**Snuff using**						
Never (reference)	1,725 (78.8)	373 (81.3)	1.00			
Former	188 (8.6)	35 (7.6)	0.861 (0.590–1.256)	0.437		
Present	254 (11.6)	43 (9.4)	0.783 (0.556–1.102)	0.161		
*Missing*	22 (1.0)	8 (1.7)				
**Drinking frequency past year**						
Never (reference)	133 (6.1)	34 (7.4)	1.00		1.000	
≤1 time/month	449 (20.5)	96 (20.9)	0.836 (0.541–1.294)	0.422	0.756 (0.450–1.270)	0.291
2–4 times/month	923 (42.2)	191 (41.6)	0.809 (0.538–1.217)	0.309	0.803 (0.494–1.306)	0.377
2–3 times/week	594 (27.1)	125 (27.2)	0.823 (0.539–1.257)	0.367	0.950 (0.568–1.587)	0.844
≥4 times/week	84 (3.8)	10 (2.2)	0.466 (0.219–0.992)	0.048	0.571 (0.235–1.384)	0.215
*Missing*	6 (0.3)	3 (0.7)				
**Drinking amount/occasion (glasses)**						
1–2 (reference)	1,115 (50.9)	249 (54.2)	1.00			
3–4	615 (28.1)	111 (24.2)	0.808 (0.633–1.032)	0.088		
5–6	208 (9.5)	44 (9.6)	0.947 (0.665–1.348)	0.764		
7–9	85 (3.9)	15 (3.3)	0.790 (0.449–1.392)	0.415		
≥ 10	23 (1.1)	4 (0.9)	0.779 (0.267–2.272)	0.647		
*Missing*	143 (6.5)	36 (7.8)				
**Physical activity at work**						
Light (reference)	1,248 (57.0)	279 (60.8)	1.00			
Intermediate	555 (25.4)	103 (22.4)	0.830 (0.648–1.063)	0.140		
Hard	309 (14.1)	56 (12.2)	0.811 (0.593–1.108)	0.189		
*Missing*	77 (3.5)	21 (4.6)				
**Physical activity during leisure time**						
Sedentary/moderate (reference)	354 (16.2)	79 (17.2)	1.00			
Training regularly	812 (37.1)	185 (40.3)	1.021 (0.763–1.366)	0.889		
*Missing*	1,023 (46.7)	195 (42.5)				

*GI, gastrointestinal; BMI, body mass index; OR, odds ratio; CI, confidence interval*.

*Physical activity at work was divided into light (mostly sitting), intermediate (walking, doing laundry), and hard (gardening, heavy lifting). Physical activity during leisure time was divided into sedentary to moderate (mostly sitting, moving without sweating) and training regularly (>30 min twice a week). Prevalence of variables was given as number and percentage. Logistic regression, adjusted for statistically significant variables found in crude OR (age, sex, BMI, education, occupation, smoking, and drinking frequency last year). P < 0.05 was considered statistically significant*.

### Association Between IBS, Gastrointestinal Symptoms the Past 2 Weeks and Sociodemographic Factors and Lifestyle Habits

**Figure 2:**
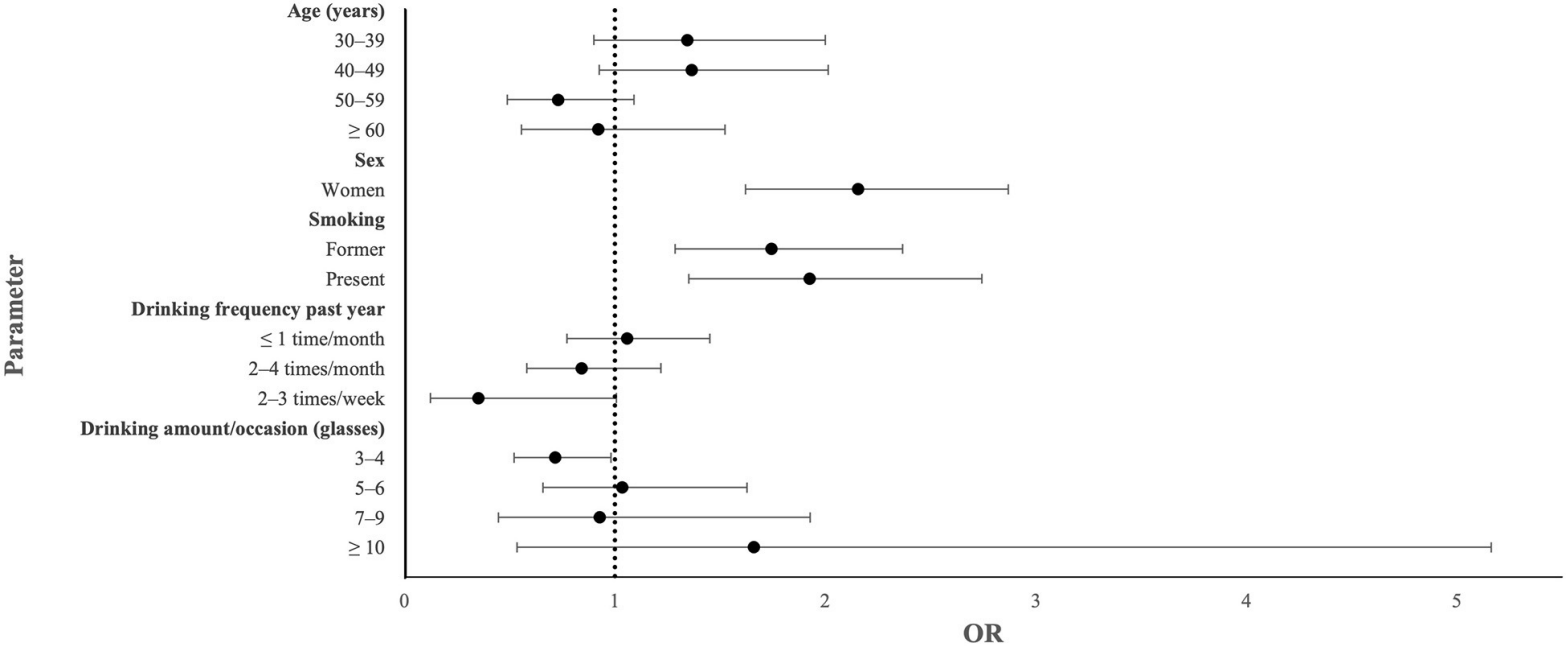
Forest plot showing odds ratio (OR) and 95% confidence interval in an adjusted model calculating sociodemographic parameters significantly associated with irritable bowel syndrome (IBS) in crude calculations.

There was a significant association between IBS and female sex (OR: 2.156; 95% CI: 1.620–2.868; *p* < 0.001), former smoking (OR: 1.744; 95% CI:1.285–2.367; *p* < 0.001), and present smoking (OR: 1.925; 95% CI: 1.352–2.742; *p* < 0.001), and an inverse association with drinking frequency of 2–3 times a week (OR: 0.352; 95% CI: 0.123–1.006; *p* = 0.051) and drinking 3–4 standard glasses per occasion (OR: 0.716; 95% CI: 0.522–0.982; *p* = 0.038) (Figure 2, Table 3).

**Figure 3:**
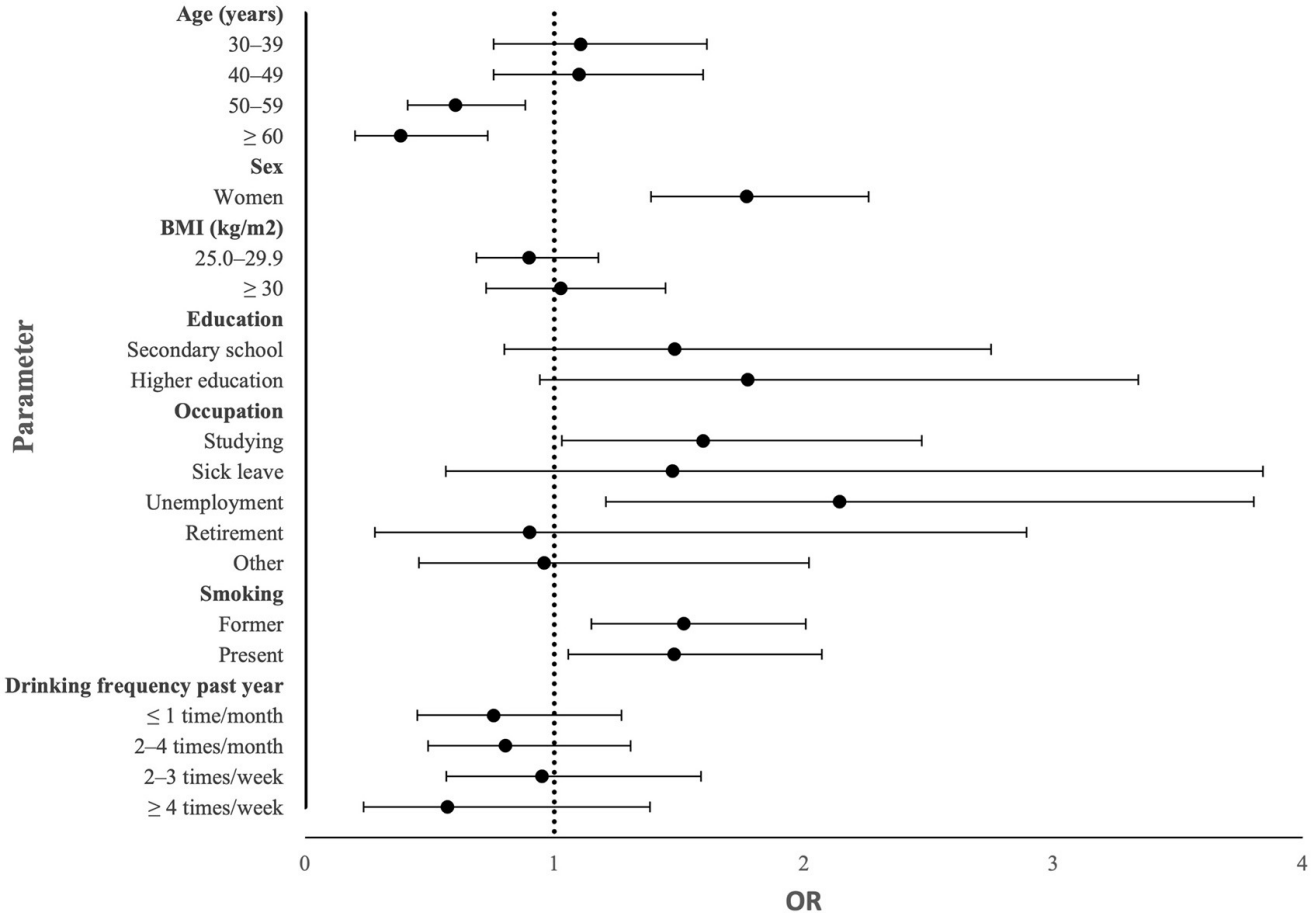
Forest plot showing odds ratio (OR) and 95% confidence interval in an adjusted model calculating sociodemographic parameters significantly associated with gastrointestinal symptoms in crude calculations.

Having GI symptoms the past 2 weeks was associated with female sex (OR: 1.770; 95% CI: 1.387–2.259; *p* < 0.001), studying (OR: 1.597; 95% CI: 1.031–2.473; *p* = 0.036), unemployment (OR: 2.143; 95% CI: 1.207–3.804; *p* = 0.009), former smoking (OR 1.518; 95% CI: 1.148–2.007; *p* = 0.003), and present smoking (OR: 1.479; 95% CI; 1.055–2.072; *p* = 0.023), whereas it was inversely associated with age 50–59 years (OR: 0.603; 95% CI: 0.412–0.883; *p* = 0.009) and age ≥ 60 years (OR: 0.384; 95% CI: 0.202–0.733; *p* = 0.004) (Figure 3, Table 4).

When examining the degree of severity of each specific GI symptom and the significant parameters from the logistic regression, i.e., age, sex, occupation, and smoking, higher age was associated with better psychological well-being. Female sex was associated with all GI symptoms, except diarrhea and vomiting and nausea, and worse psychological well-being. Present smoking was associated with more symptoms and worse psychological well-being. Former smoking was only associated with bloating and flatulence (β: 7.531; 95% CI: 1.370–13.692; *p* = 0.017). Studying was associated with more abdominal pain and bloating and flatulence but improved psychological well-being. Sick leave and unemployment were associated with severe symptoms and poor psychological well-being in comparison to working (Table 5).

**Table 5:** Associations between age, sex, occupation, and smoking and specific symptoms.

**Variables**	**β**	**95% CI**	***P*** **-value**
**Age**			
Psychological well-being	−2.029	−26.48 to −1.410	<0.001
**Sex (men reference)**			
Abdominal pain	9.859	4.820–14.897	<0.001
Constipation	15.226	9.874–20.579	<0.001
Bloating and flatulence	15.720	10.253–21.187	<0.001
Influence on daily life	10.630	5.088–16.172	<0.001
Psychological well-being	4.395	2.684–6.105	<0.001
**Present smoking**			
Abdominal pain	8.945	2.448–15.442	0.007
Diarrhea	11.971	5.180–18.761	0.001
Constipation	8.043	1.259–14.827	0.020
Bloating and flatulence	7.883	0.918–14.848	0.027
Vomiting and nausea	7.722	1.377–14.068	0.017
Influence on daily life	8.652	1.558–15.747	0.017
Psychological well-being	5.842	3.356–8.329	<0.001
**Occupation**			
***Working*** (reference)			
***Studying***			
Abdominal pain	35.702	10.137–61.267	0.006
Bloating and flatulence	31.081	2.590–59.573	0.033
Psychological well-being	−6.875	−12.449 to −1.300	0.016
***Sick leave***			
Abdominal pain	27.802	4.897–50.707	0.017
Constipation	29.813	5.960–53.667	0.014
Bloating and flatulence	26.664	3.324–50.005	0.025
Vomiting and nausea	28.373	9.710–47.037	0.003
Influence on daily life	35.268	13.840–56.695	0.001
Psychological well-being	23.170	15.200–31.140	<0.001
***Unemployment***			
Constipation	11.719	2.582–20.855	0.012
Vomiting and nausea	17.324	9.403–25.245	<0.001
Psychological well-being	5.280	1.964–8.595	0.002
***Retirement***			
Psychological well-being	12.477	7.263–17.692	<0.001
***Other***			
Vomiting and nausea	17.290	1.069–33.511	0.037

*β, beta-value; CI, confidence interval. Symptoms the past 2 weeks were assessed by the visual analog scale for irritable bowel syndrome (VAS-IBS) where 0 mm means no symptoms and 100 mm means maximal symptoms (20). Generalized linear model, using variables significantly associated with GI symptoms in the logistic regression. Age groups was calculated as a continuous variable. P < 0.05 was considered as statistically significant*.

No statistically significant association**s** were found between IBS or GI symptoms the past 2 weeks and BMI groups, education, marital status, snuff use, physical activity at work, or physical activity during leisure time (Tables 3, 4).

### Associations Stratified by Sex

When examining the differences between sex in the present cohort, women had lower BMI group (*p* < 0.001), higher education degree (*p* < 0.001), were more often sick and less often unemployed or retired (*p* = 0.008), less often snuff users (*p* < 0.001), drank alcohol less often (*p* < 0.001) and in less amounts (*p* < 0.001), and had less physical activity at work (*p* < 0.001) in comparison to men. Smoking prevalence did not differ between sexes (*p* = 0.093). In the interaction analysis, sick leave was the only parameter which interacted with sex for having IBS (pi = 0.021).

In men, there was a significant association between IBS and being 30–49 years old and being unemployed or other (Table 6). Regarding GI symptoms the past 2 weeks, the only association observed was with being unemployed (Table 7). In women, there was a significant association between IBS and being present smoker (Table 6). GI symptoms were associated with former smoking and inversely associated with age ≥ 50 years and intermediate physical activity at work (Table 7).

**Table 6:** Associations between sociodemographic factors, lifestyle habits and IBS stratified by sex.

**Variables**	**Men**		**Women**	
	**OR and 95% CI**	***P*** **-value**	**OR and 95% CI**	***P*** **-value**
**Age (years)**				
<30 (reference)	1.000			
30–39	3.050 (1.355–6.863)	0.007		
40–49	2.773 (1.231–6.241)	0.014		
50–59	1.163 (0.492–2.753)	0.731		
≥60	1.486 (0.472–4.674)	0.498		
**Occupation**				
Working (reference)	1.000			
Studying	1.161 (0.364–3.705)	0.801		
Sick leave	4.063 (0.423–38.991)	0.224		
Unemployment	5.505 (2.293–13.218)	<0.001		
Retirement	1.115 (0.203–6.112)	0.900		
Other	4.936 (1.656–14.713)	0.004		
**Smoking**				
Never (reference)			1.000	
Former			1.254 (0.885–1.776)	0.203
Present			1.591 (1.069–2.367)	0.022
**Snuff using**				
Never (reference)			1.000	
Former			1.892 (0.883–4.051)	0.101
Present			1.122 (0.505–2.492	0.777

*IBS, irritable bowel syndrome; BMI, body mass index; OR, odds ratio; CI, confidence interval. Logistic regression adjusted for statistically significant variables found in crude OR (age and occupation in men and smoking and snuff in women). P < 0.05 was considered statistically significant*.

**Table 7:** Association between sociodemographic factors, lifestyle habits and GI symptoms past 2 weeks stratified by sex.

**Variables**	**Men**		**Women**	
	**OR 95% CI**	***P*** **-value**	**OR 95% CI**	***P*** **-value**
**Age (years)**				
<30 (reference)	1.000		1.000	
30–39	1.420 (0.793–2.543)	0.238	0.944 (0.582–1.531)	0.817
40–49	1.299 (0.718–2.347)	0.387	0.959 (0.603–1526)	0.859
50–59	0.734 (0.404–1.337)	0.312	0.519 (0.324–0.830)	0.006
≥ 60	0.518 (0.188–1.427)	0.203	0.296 (0.125–0.701)	0.006
**BMI (kg/m^2^)**				
<25 (reference)	1.000			
25.0–29.9	0.816 (0.533–1.249)	0.349		
≥ 30	0.974 (0.570–1.662)	0.922		
**Occupation**				
Working (reference)	1.000	1.000		
Studying	1.346 (0.655–2.768)	0.419	1.317 (0.754–2.299)	0.333
Sick leave	–	–	0.647 (0.140–2.985)	0.577
Unemployment	3.612 (1.675–7.788)	0.001	1.785 (0.731–4.360)	0.203
Retirement	0.442 (0.050–3.880)	0.461	1.376 (0.256–7.396)	0.710
Other	1.857 (0.592–5.824)	0.289	0.719 (0.270–1.914)	0.509
**Smoking**				
Never (reference)			1.000	
Former			1.452 (1.020–2.067)	0.038
Present			1.481 (0.964–2.274)	0.073
**Physical activity at work**				
Light (reference)			1.000	
Intermediate			0.621(0.436–0.885)	0.008
Hard			0.688 (0.404–1.172)	0.169

*GI, gastrointestinal; BMI, body mass index; OR, odds ratio; CI, confidence interval*.

*Physical activity at work was divided into light (mostly sitting), intermediate (walking, doing laundry), and hard (gardening, heavy lifting). Logistic regression adjusted for statistically significant variables found in crude OR (age, BMI, and occupation, in men and age, occupation, smoking, and physical activity at work in women). P < 0.05 was considered statistically significant*.

## Discussion

The main findings in the present population-based study were that IBS and GI symptoms the past 2 weeks were associated with female sex and smoking. Further, IBS was inversely associated with drinking moderate amount of alcohol and GI symptoms were associated with studying and unemployment and inversely associated with higher age. In men, IBS was associated with middle-age and both IBS and GI symptoms were associated with being unemployed. In women, IBS and GI symptoms were associated with present and former smoking, respectively, and GI symptoms were also inversely associated with higher age and intermediate physical activity at work. Thus, our hypothesis that smoking and snuff using have similar adverse impact on the GI symptoms and that higher alcohol consumption, living alone, lower BMI, lower education, and lower physical activity are associated with IBS or more symptoms could not be confirmed.

The female dominance in IBS is well-described in several studies (2, 7, 13), and is also confirmed in the present study of the general population. The age was lower in the subjects with IBS and GI symptoms than in those without symptoms. Calculations stratified by sex showed that men were more prone to suffer from IBS in the middle-age, and women were less prone to have GI symptoms when they were elder.

In line with a previous population-based study in elderly, participants with IBS and GI symptoms had lower BMI than subjects without IBS and GI symptoms (11). However, when performing logistic regression and adjusting for confounders, no association between IBS or GI symptoms and BMI groups could be found. These differences in calculations may explain contradictory results from other studies regarding BMI and IBS (9–11). Neither marital status nor education did have any effect on IBS or GI symptoms, although this has been described in previous studies (8, 9).

Both current and former smoking were associated with IBS and GI symptoms, which is in line with previous research (13, 14, 23, 24). However, when examining the severity of individual GI symptoms, only present smoking was associated with the symptoms, except bloating and flatulence. The association with smoking was slightly stronger in this cohort than in a cohort of elder subjects from the general population (13). Smoking has been shown to be able to produce acute analgesic effect, which may improve abdominal pain and lead to continued smoking and poor motivation for smoking cessation (25). However, other studies have described how smoking may induce both visceral and peripheral hypersensitivity (26), which is supported by the strong association between abdominal pain and current smoking in the present study. Furthermore, IBS is associated with both diarrhea and/or constipation. Smoking has been found to delay the gastric emptying of solids in non-smokers, independently of nicotine (27). On the other hand, acute cigarette smoking in habitual smokers delayed mouth-cecum transit time, most likely due to nicotine (28). Transdermal nicotine application in non-smokers has been found to accelerate the transit time in the colon, sigmoideum, and rectum, and thereby decrease the total colon transit time (29). Male smokers, in contrast to female smokers, have prolonged colonic transit time compared to non-smokers in the same sex (30). These effects of smoking on GI motility may explain why smokers suffer from diarrhea and constipation. The association between smoking and functional abdominal pain may thus partly be explained by these alterations in motility (31). Although the role of smoking on gut microbiota is only rudimentary examined and may be influenced by weight and other lifestyle factors, studies suggest that smoking induces specific changes in the microbiome; changes that are reversed after smoking cessation (32). These changes of microbiota may also be of importance for the experience of IBS and GI symptoms (2, 32).

The difference between smoking and snuff using in association with IBS may depend on that cigarette smoking contain multiple toxins and cacogenic substances affecting the GI tract, which are not found to the same extent in snuff (33). Furthermore, the number of snuff users were lower, which may affect the calculations. When stratifying the results by sex, women but not men, had an association between smoking and both IBS and GI symptoms, even though there was no dependency found between sex and smoking in this cohort. The reason for this could be that the number of smoking men with IBS was too few. Generally, women tend to smoke more than men, while men tend to use snuff more (34). The latter was in line with the snuff habits in our cohort, but the smoking habits were equal between sexes. Snuff prevents smoking initiation and is used as a cessation aid, making the users less likely to smoke (34, 35). This could be seen in this cohort as well, where there was a dependency between smoking and using snuff. Even though snuff contains tobacco and nicotine, it has a less negative overall impact on the health (36, 37).

Binge drinking has previously been reported to be associated with GI symptoms the day after drinking (12, 15), while light to moderate drinking did not show any association with symptoms (12, 14, 15). Other studies have shown that 12–17% of those with IBS report alcohol intolerance, and 50% restrain themselves from drinking (38–40). This can explain why the IBS prevalence was higher in non-users of alcohol (9). On the other hand, there is a strong association between poor psychological well-being and severe GI symptoms (1, 2), and people with more pain could also have a reason for more drinking, as alcohol is associated with calming and numbing effects (22). The present study showed weak inverse association between IBS and an alcohol consumption of 3–4 glasses per occasion. Taken together, the conclusion can be drawn that alcohol abuse is not responsible for the great burden of IBS or GI symptoms in the population.

Being unemployed was associated with GI symptoms, especially in men. Unemployment and “other” were also associated with IBS in men. The group other is a bit vague, but the question states that they are not working, but doing something else. Sick leave and unemployment were associated with more severe symptoms and impaired psychological well-being. This is in line with previous research showing a high degree of unemployment and absence from work in patients with self-reported IBS (41). While being unemployed, a social stigma can arise, and both being without a job and the fear of stigma can increase the stress levels (42). All type of stress is a well-known factor associated with FGID (43). Research has found that men have worse mental health than women when they are unemployed and living with somebody else. This could be due to that men traditionally are considered as the family's main breadwinner, and to provide this is their primary responsibility (44). In line with this, the subjective social status relative to the others in the same country mediates the association between occupational status, mental health, and stress (45).

There was no difference in physical activity between subjects with IBS or GI symptoms and those without symptoms. Nevertheless, several studies have shown that physical inactivity is associated with FGID (11), and that physical activity may decrease symptoms (46). Thus, although physical activity has been proven to be of benefit for these patients, inactivity could probably not explain the development of the disease in the general population. The higher degree of education in women may explain the lower physical activity at work for women.

While comparing different studies, it is important to understand that there are variations in IBS definitions (e.g., different Rome criteria), affecting the inclusion criteria and cohort compositions (3, 4). Therefore, we chose to include the VAS-IBS recording all GI symptoms independent of classifications. This questionnaire is valid throughout decades, since it only assesses the symptoms, without any defined combinations of symptoms (20). The recruitment process of participants is also important. This study included participants originating from the general population and not from a hospital, which might otherwise be a common source. Also, the participants' age can vary, where previous research focuses more on younger participants (47), while the age in this cohort is more evenly spread in different age groups.

The major strength of this thesis is the large cohort of patients from the general population with validated questions of specific GI symptoms. It is also a strength that associations with snuff habits have been examined. One limitation was that the questionnaire was extensive, and participants may suffer from reading fatigue with several questions to answer. Selection of offspring to MCC-CC may render recall bias. Another limitation with the source material is the question regarding IBS since the Rome questionnaire was not completed depending on its size (48). However, IBS is a well-known description of GI symptoms in the society, and people are familiar with the symptoms and criteria (41). There is also a risk of missing newly debuted cases when asking for symptoms in the past, e.g., 6–12 months, which means that some of the data could be wrongly categorized. On the other hand, just to assess the GI symptoms during the past 2 weeks is also a limitation. When grouping by occupation, those who had selected multiple choices about their occupation was marked as missing, because it was not clearly stated their primary activity or distribution. The cohort was too small in some cases, and some groups had to be merged to get a reasonable volume to compare, which makes it possible that some details were lost.

In conclusion, smoking seems to be a lifestyle habit which is clearly associated with IBS. Confirmation of this finding in several studies suggest that prevention of subjects to start smoking, and smoking cessation in smokers, are lifestyle habits important to promote. An awareness of the risk for unemployed men to suffer from IBS and GI symptoms is important to remember in daily clinical praxis. However, other sociodemographic factors and lifestyle habits assessed in this study are not the main reason for subjects to suffer from IBS and GI symptoms in the general population.

## Data Availability Statement

The raw data supporting the conclusions of this article will be made available by the authors, without undue reservation.

## Ethics Statement

The studies involving human participants were reviewed and approved by Regional Ethics Review Board in Lund (2012/594). The patients/participants provided their written informed consent to participate in this study.

## Author Contributions

DN and BO were responsible for study design, acquisition of data, statistical analysis, interpretation of data, and drafting the manuscript. DN was responsible for visualization. BO obtained funding. Both authors contributed to the article and approved the submitted version.

## Conflict of Interest

The authors declare that the research was conducted in the absence of any commercial or financial relationships that could be construed as a potential conflict of interest.

## Publisher's Note

All claims expressed in this article are solely those of the authors and do not necessarily represent those of their affiliated organizations, or those of the publisher, the editors and the reviewers. Any product that may be evaluated in this article, or claim that may be made by its manufacturer, is not guaranteed or endorsed by the publisher.
